# Expanding the Repertoire of Biomarkers for Alzheimer’s Disease: Targeted and Non-targeted Approaches

**DOI:** 10.3389/fneur.2015.00256

**Published:** 2015-12-16

**Authors:** Douglas Galasko

**Affiliations:** ^1^Department of Neurosciences, Shiley-Marcos Alzheimer’s Disease Research Center, University of California, San Diego, La Jolla, CA, USA

**Keywords:** Alzheimer’s disease, biomarker, biofluid, amyloid, tau, synapse, proteomics

## Abstract

The first biofluid markers developed for Alzheimer’s disease (AD) used targeted approaches for discovery. These initial biomarkers were directed at key protein constituents of the hallmark brain lesions in AD. Biomarkers for plaques targeted the amyloid beta protein (Aβ) and for tangles, the microtubule-associated protein tau. Cerebrospinal fluid levels of Aβ and tau have excellent diagnostic utility and can be used to monitor aspects of therapeutic development. Recent research has extended our current concepts of AD, which now include a slow buildup of pathology during a long pre-symptomatic period, a complex cascade of pathological pathways in the brain that may accelerate once symptoms develop, the potential of aggregated proteins to spread across brain pathways, and interactions with vascular and other age-associated brain pathologies. There are many potential roles for biomarkers within this landscape. A more diverse set of biomarkers would provide a better picture of the staging and state of pathological events in the brain across the stages of AD. The aim of this review is to focus on methods of biomarker discovery that may help to expand the currently accepted biomarkers. Opportunities and approaches for targeted and non-targeted (or −omic) biomarker discovery are highlighted, with examples from recent studies. How biomarker discoveries can be developed and integrated to become useful tools in diagnostic and therapeutic efforts is discussed.

## Introduction

Biomarkers have many potential uses in Alzheimer’s disease (AD), related neurodegenerative disorders and brain aging. Initial efforts to develop diagnostic biomarkers for AD were focused on the hallmark pathological lesions of senile plaques and neurofibrillary tangles. Amyloid beta-protein (Aβ), an integral component of plaques, and the microtubule-associated protein tau, the major protein found in tangles, were detected in cerebrospinal fluid (CSF). Sensitive enzyme-linked immunosorbent assays (ELISAs) were developed to selectively detect pathogenic forms of Aβ (Aβ42) and tau, with the later advent of assays for phosphorylated forms of tau (P-tau) (1–3). Different phosphor-epitopes of tau have been identified in CSF and are increased in AD, including tau phosphorylated at threonine181 (the form most commonly measured), serine 199, and serine 231 (4). Increased levels of P-tau are more specific for AD than other dementias and may add value in differential diagnosis (4, 5). The profile of decreased Aβ42 and increased total tau and P-tau in CSF has high diagnostic value for AD (6) and has been a mainstay of AD biomarker research. Changes in CSF biomarkers are apparent in early symptomatic stages of AD, such as mild cognitive impairment (MCI) (7), and also occur pre-symptomatically (8). In these settings, the core biomarkers can provide prognostic information, for example, which patients with MCI may progress to AD dementia (7, 9–11). Also, studies have shown that patients with MCI or AD with higher baseline levels of CSF tau or P-tau (12, 13), and more recently higher baseline levels of the postsynaptic protein neurogranin (14) may show more rapid progression. This indicates the value of CSF biomarkers for predicting progression, e.g., for prognosis in preclinical stages of AD. Many forms of A-beta exist in CSF, and profiling N-terminal truncated forms was shown to increase prognostic value in MCI in one study (15).

**Figure 1:**
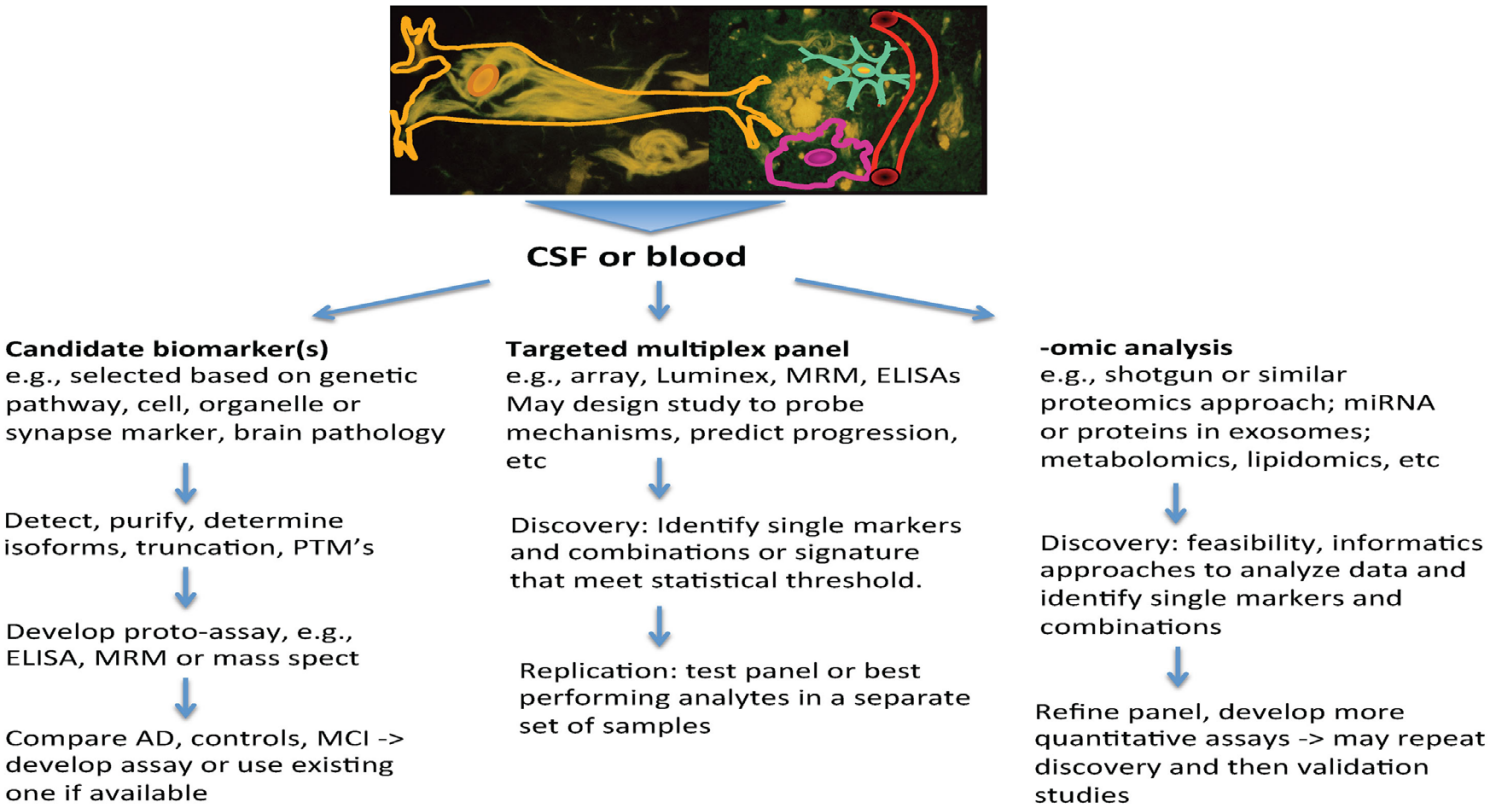
**Biomarker discovery for Alzheimer’s disease – approaches**.

Several themes that have emerged from AD research highlight the increased need for biomarkers, and also set the stage for how they may be used. First, AD is now viewed as a chronic and slowly progressive disorder, with a long buildup of pathology that precedes symptoms by a decade or longer (16). Also, among people with late-onset AD, autopsy studies highlight the frequent co-occurrence of other brain pathologies, such as vascular changes (macro-infarcts, lacunes and micro-infarcts, amyloid angiopathy, arteriosclerosis, and microbleeds) and other protein aggregates (e.g., alpha-Synuclein and TDP43) (17, 18). These may contribute to dementia and can be difficult to detect during life. In patients with atypical presentations, such as younger onset of dementia, the clinical picture may not be clear, and biomarkers can provide pointers to underlying pathology. Finally, treatment interventions for AD are shifting to earlier intervention, including stages of prodromal AD, where symptoms are mild, and most recently to prevention studies, where cognition falls within normal limits. Biomarkers have valuable roles to play in this pre-symptomatic stage to provide measures that may guide therapeutics. By measuring several biomarkers in CSF through individual or multiplex assays, it may be possible to index a number of biochemical processes in the brain that are informative about AD and related neurodegenerative disorders simultaneously. This enhances the value of CSF sampling. This review will summarize the potential roles for biomarkers and how approaches to biomarker discovery can help to build a pipeline that will address these needs and inform risk assessment, diagnosis, and treatment (Figure 1).

## Sources of Fluid Biomarkers

The most obvious source of biomarkers relevant to the brain is CSF, which bathes the brain and spinal cord. CSF biomarkers reflect overall brain biochemistry, and processes such as neuronal damage, synapse loss, and inflammation may result in detectable biomarker changes in CSF if they are extensive enough. CSF is sampled through the lumbar space and may have different concentrations of analytes compared to the ventricular CSF. Typically, analytes are more concentrated in lumbar CSF, as noted for Aβ40, Aβ42, and tau (19). The question of concentration gradients within the lumbar CSF arises for many analytes and needs to be studied – this is not a major problem for Tau, P-tau, and Aβ42. Blood derivatives, such as plasma and serum, are easier to access than CSF, but typically reflect the body as a whole. If a brain-specific protein crosses into the blood, it may be subject to dilution, the action of proteases, and clearance by the liver and kidney, rendering it difficult to detect. As a further complication, systemic features of AD, such as weight loss or lower physical activity may result in subtle changes in blood biomarker levels. These are many of the reasons why it has been extremely difficult to identify a blood biomarker that directly reflects the state of neurodegeneration (20, 21).

There are other questions or areas where blood biomarkers may have utility. Some plasma or serum analytes may relate to traits that predispose to neurodegeneration, for example, biomarkers that may be influenced by susceptibility genes. If age or environmental risk factors related to dementia have systemic effects, then these may be evident through the analysis of blood biomarkers. Blood biomarkers are particularly helpful as measures of drug levels and can provide peripheral indices of target engagement. Blood cells, e.g., lymphocytes or leukocytes, may be used to derive immune signatures or measures of RNA expression that may be indices of susceptibility for AD. Plasma and blood biomarkers are influenced by genetic factors and a wide spectrum of environmental factors, for example, diet, systemic illness, and physical activity. A recent paper studied over 300 plasma analytes longitudinally in twins, and identified variability that could be attributed to all of these factors. These findings suggest that a search for peripheral markers for AD may be extremely complicated, because in addition to these variables, aging is yet another factor that may impact on levels of peripheral markers. Plasma levels of Aβ, including ratios between different forms of Aβ (such as the ratio of Aβ42/Aβ40) have been inconsistent across studies, are only weakly correlated with CSF levels of Aβ or with markers of amyloid brain imaging, and although they may have some predictive value for the development of AD, this is relatively low [reviewed in Ref. (22)]. Peripheral issue may be a source of pathological proteins if there are systemic features of a neurodegenerative disease. This has been identified in Parkinson’s disease (PD), where nerve endings can be stained for abnormal forms of alpha-synuclein in skin and salivary gland biopsy (23).

Regardless of whether a biomarker is measured in blood, CSF, or in biopsy material, data that shed light on how the biomarker is produced, released, cleared, and metabolized should be sought. To understand the biomarker comprehensively, it may require data from cell, model organism, and animal studies, as well as human biofluids and postmortem tissue. A recent development is the ability to study kinetics of CSF and plasma analytes by administering stable isotopes intravenously or orally to human subjects (24, 25). Examining the relationships between different types of biomarkers can also inform about pathogenetic processes, for example, by correlating biofluid biomarker changes with neuroimaging markers. This also allows modeling of when the biomarker becomes abnormal and how it changes during the early course of AD (26).

## Expanded Roles for Biomarkers in AD

There are many potential roles of biomarkers for AD and neurodegenerative disorders (Table 1). New biomarker discovery efforts need to take into consideration the current landscape of AD diagnosis and treatment efforts. The clinical diagnosis of typical AD by experts is often highly accurate; therefore, diagnostic biomarkers should be sensitive enough to help in early diagnosis, e.g., at stages of MCI or prodromal AD (27, 28). Because the sensitivity of CSF Aβ42, tau, and P-tau to discriminate prodromal AD from cognitively normal individuals is high, it may be challenging for additional biomarkers to improve on this. The differential diagnosis of unusual or atypical cases is a situation where biomarkers may clearly augment clinical judgment. Evaluating whether non-AD pathology may be present is an important question, particularly in elderly individuals with cognitive problems, and additional biomarkers could be helpful if they inform about processes, such as alpha-Synuclein, TDP-43, or vascular brain pathology. Mixed pathology is often present in the brains of elderly individuals with dementia, and a biomarker panel that allowed clear prediction of the types of underlying pathology would be useful.

**Table 1:** **Roles for fluid biomarkers in Alzheimer’s disease**.

Roles in diagnosis, staging, and prognosis	Examples of biofluid markers	Comments	Reference
Screening or diagnosis with a blood test	Plasma Aβ42/Aβ40	Weak predictive value	Toledo et al. (22)
		Not diagnostic	
	Multi-analyte test panels, e.g., phospholipids	Initial good discrimination, needs replication	Mapstone et al. (29), Sattlecker et al. (30)
	Multi-analyte protein screening (e.g., Somascan)		
	Plasma t-Tau	Slight increase in AD; much overlap	Zetterberg et al. (31)
	Auto-antibody profile; peptoids	Needs replication	Reddy et al. (32), Nagele et al. (33)
Diagnosis of AD vs. control	CSF Aβ42, t-Tau, P-Tau	Validated extensively	Skillbäck et al. (5)
Diagnosis of AD pathology at prodromal or MCI stage	CSF Aβ42, t-Tau, P-Tau	Validated extensively	Mattsson et al. (7)
Differential diagnosis	CSF Aβ42, t-Tau, P-Tau	Validated extensively	Skillbäck et al. (5)
Predict progression, e.g., from control to AD, MCI to AD, and rate of progression in AD	High levels of CSF t-Tau, P-Tau	Validated extensively	Shaw et al. (10)
	Ratios e.g., Aβ42/t-Tau Low CSF Aβ42	Aβ42 alone does not predict AD progression rate	
	N-terminal truncated CSF Aβ42	MCI progression	Vanderstichele et al. (15)
	Neurogranin	MCI progression	Kvartsberg et al. (14)
	YKL40	MCI progression, control progression when combined with Aβ42	Craig-Schapiro et al. (34)
	Visinin-like protein-1	MCI progression; control progression especially when combined with Aβ42	Tarawneh et al. (35)
Diagnosis of non-AD disorders	Few specific markers, but ratios and patterns help, e.g., very high tau in CJD; ratio of P-tau/T-tau in FTLD-tau; high neurofilament-L levels in vascular cognitive impairment, PSP and FTLD		Skillbäck et al. (36), Hu et al. (37)
	α-Synuclein	Decreased in Parkinson’s but sensitivity is not diagnostically useful	Parnetti et al. (38)
Understand genetic and other risk factors	CSF t-Tau, P-tau, Aβ42 ApoE protein, clusterin, inflammatory cytokines TREM-2	Genes or SNPs associated with AD genetic risk may relate to levels of CSF biomarkers	Cruchaga et al. (39)
Markers of pathobiology	Neurogranin, SNAP-25	Many other potential pathways and processes may be reflected in CSF	
Synaptic damage	Visinin-like-protein-1; t-Tau		
Neuronal damage	NFL		
Axonal tracts	Cytokines, chemokines, c3, YKL-40		
Inflammation			
Blood–brain barrier integrity and small vessel CNS ischemia	CSF: serum albumin ratio, IgG index, MMP 2, 3, and 9; NFL	Altered in vascular cognitive impairment	Rosenberg et al. (40)

**Roles in therapeutics**	**Examples**	**Comments**	**Reference**

Preclinical drug development	Aβ42, t-Tau, P-Tau	Can be used to evaluate compounds in cell and animal models	Liu et al. (41), Jeppsson et al. (42)
	sAPPα and sAPPβ		
	Oligomeric forms of Aβ and Tau		
Target engagement in pharmacodynamic studies	Aβ40 and 42 in CSF and plasma for gamma-secretase inhibitors	Has helped with dose finding	Fleisher et al. (43)
	sAPPα, sAPPβ, and Aβ for BACE inhibitors		
Detailed studies of synthesis and clearance	SILK studies for Aβ metabolism	Used to characterize detailed pharmacodynamics of anti-Aβ therapeutics	Bateman et al. (24)
Patient selection or stratification	CSF Aβ42, t-Tau, P-tau	Enroll patients with AD signature	Coric et al. (44)
Identify toxicity	Increases in CSF biomarkers of neuronal damage or of inflammation		Van Gool et al. (45)
Provide biological support for treatment effect	Changes in CSF Aβ-related biomarkers indicating target engagement and of t-Tau, P-Tau, or synaptic markers such as neurogranin in a direction indicating reduction of neurodegeneration	Changes in a group of markers could support anti-amyloid therapeutics	

*This table is not intended as a comprehensive listing, but shows representative biomarkers that can aid in diagnostic or therapeutic efforts. The biomarkers in this table are discussed in the text. The majority of markers are proteins discovered through candidate approaches, but there is room for an expanded suite of markers using diverse discovery approaches to improve our understanding of AD*.

*t-Tau, total tau levels; α-Syn, alpha-Synuclein; sAPP, secreted amyloid protein precursor; NFL, neurofilament light; TREM2, triggering receptor expressed on myeloid cells 2; MBP, myelin basic protein; MMP, matrix metalloprotease; BACE, beta-site APP cleaving enzyme 1; SILK, stable isotope kinetic labeling; CJD, Creutzfeld–Jacob disease; PSP, progressive supranuclear palsy; FTLD, fronto-temporal lobar degeneration*.

Therapeutic efforts for AD are shifting to earlier intervention, including studies of secondary prevention, and even primary prevention in people with genetic predisposition. Potential uses of CSF biomarkers in clinical trials for AD and PD were recently reviewed in detail (46). Neuropathology and clinical research have shown that there are preclinical stages of AD during which amyloid and tau pathology accumulates, before the onset of memory decline (47). This provides an opportunity to start treatment interventions with the goal of delaying the onset of AD. Changes in biomarkers may provide a clearer early readout from prevention studies than changes in cognitive measures. Biomarkers are critical to identifying the presence of amyloid or tau brain pathology in this situation. For studies of early intervention, screening biomarkers, e.g., blood tests, that can improve the likelihood of detecting pathological brain changes through a more definitive test, such as molecular brain imaging and lumbar puncture, would be a great asset.

Biomarkers may help to improve the understanding of risk factors and mechanisms of disease. One would expect that causative or susceptibility genetic factors should be easy to link to biomarkers in biofluids. This has only been demonstrated in a few instances. For example, in AD, the APOE e4 allele has not yet been associated with a unique biomarker profile but modulates levels of the ApoE protein (48, 49). Inflammation plays a role in AD and other neurodegenerative disorders, and genetic variants related to the TREM2 gene increase the risk of AD and other dementias (50). In CSF, levels of a secreted soluble form of TREM were recently found to be decreased in AD (51). Other inflammatory biomarkers, such as secreted cytokines and chemokines, are unchanged or slightly increased in CSF in AD (52, 53). CSF biomarkers have been used as endophenotypes to discover genetic variants related to their levels, for example, CSF tau in AD (39), and CSF biomarkers related to inflammation (54). Genetic forms of non-AD dementia have provided clues for novel biomarkers. For example, inherited forms of fronto-temporal dementia (FTD) due to mutations in the progranulin gene result in haplo-insufficiency with decreased production of granulin. Correspondingly, levels of granulin in plasma and CSF are markedly (and diagnostically) decreased (55). Burgeoning research on AD pathology has identified abnormalities in many biological processes, and it is likely that many pathogenic steps and events are occurring in a cascade (56). It may be feasible to develop biomarkers that can help to track many of these events, for example, microglial activation, inflammation, synaptic damage, and dysfunction (discussed later). This approach, together with neuroimaging methods, offers an opportunity to build a more complete picture of neurodegeneration in living patients at different stages of disease.

There are many potential therapeutic applications of biomarkers in AD. These typically have involved targeted biomarkers. During preclinical development, screening for gamma-secretase inhibitors and modulators and BACE inhibitors in cell and animal models have obtained their readout by using assays for the same secreted forms of Aβ that are used in AD diagnosis (57–59). These assays can be further applied to animal models and in human studies to identify target engagement and pharmacodynamic effects. A more detailed application is through CSF catheter placement to sample CSF during 24–36 h. This has been extended using stable isotope labeling kinetics (SILK) to estimate the fractional production and clearance rates of Aβ from CSF (24). In clinical trials, CSF biomarkers may be used to select patients or to stratify treatment. For example, in trials that aim to enroll patients with MCI due to AD, requiring a baseline CSF biomarker profile can increase confidence that the study population has symptoms due to AD rather than other causes. Target engagement may be demonstrated for certain types of amyloid-related interventions, in particular, secretase inhibitors. For example, gamma-secretase inhibitors that were studied in human clinical trials (43) and Beta-secretase inhibitors (60) showed robust effects in decreasing secreted forms of APP as well as Aβ in early phase studies, and the gamma-secretase inhibitor semagacestat showed plasma biomarker evidence of target activation in a phase 3 trial (61). Mass spectrometry (MS) characterization has identified a specific Aβ peptide signature after BACE inhibitor treatment (60). However, it is more challenging to show target engagement by antibodies directed against Aβ, because these bind Aβ and alter its levels in CSF and plasma. As novel drug targets are identified, efforts to identify companion biomarkers that help to identify immediate and downstream effects of drug action should be pursued.

Changes in levels of tau and P-tau in CSF have been examined as prototypic AD biomarkers of neurodegeneration or neuronal damage, with the hypothesis that neuroprotective or disease-modifying drug effects may result in a decrease of these markers. It is likely that profiling biomarkers more broadly could be more informative. For example, biomarkers that index aspects of pre- and postsynaptic change, microglial activation, and astrocytic responses combined with neuroimaging could provide greater insights into the dynamics and interactions of neurons and glial cells in response to interventions. In efforts to make a claim to support drug efficacy, biofluid biomarkers are expected to play a supporting rather than a primary role. For example, if one of the effects of a drug treatment is to slow neurodegeneration enough to produce a meaningful cognitive readout, biomarker changes could be used to identify which disease-related pathways have been affected. To better understand events during neurodegeneration or disease progression, further exploration using non-targeted −omic approaches is worth pursuing. A complicated situation arises if biomarker changes are present in the absence of an appropriate clinical readout; this could indicate that the drug hit its target and influenced biomarkers but this is ineffective clinically, or that the changes in the biomarker are ambiguous. For example, CSF P-tau levels have been shown to decrease significantly in patients who received bapineuzumab, with a trend for total tau to decrease, but this did not correlate with clinical efficacy (62).

## Approaches to Discover Biomarkers in Biofluids

Protein and peptide biomarkers in biofluids have formed the mainstay of clinical diagnostic tests in AD and other neurodegenerative disorders. As discussed above, despite over two decades of research, we have identified only a small number of fluid biomarkers for AD. The currently available biomarkers of CSF Aβ, tau, and P-tau have problems with measurement and standardization issues (63) that have hindered their routine and widespread use. The development of quality standards, a MS assay, and second-generation assays for these analytes are likely to improve this situation. As yet there are no established biomarkers for other neurodegenerative disorders and for vascular cognitive impairment. In view of the complexity of AD, the coexistence of mixed pathology in late-onset dementia, and the increasing emphasis for early diagnosis of AD and other neurodegenerative disorders, the search for additional biomarkers is highly warranted. One challenge is that CSF and plasma both contain proteins whose concentration spans several orders of magnitude, and almost all other proteins are overshadowed in concentration by albumin. Methods to identify novel biomarkers, in particular, proteomics, have improved, allowing post-translational modifications to be sought, and low abundance proteins (members of the “deep proteome”) to be detected. Two main strategies for biomarker discovery have emerged, namely, targeted or candidate biomarker discovery, and multiplex or −omic approaches.

### Targeted Approaches to Identify and Develop Protein and Peptide Biomarkers

The search for targeted or candidate biomarkers for AD met with significant early successes. Based on the expectation that abnormal forms of Aβ and tau could be found in CSF, methods to detect forms of these proteins in CSF and plasma were developed. Many important and complex steps have been involved in understanding and translating these hallmark AD biomarkers. To start, assays that selectively detected the longer and more aggregation-prone form of Aβ, Aβ42, were required. Total levels of Aβ in CSF were unchanged in AD, and the paradox that levels of Aβ42 were selectively decreased in CSF in AD (1) has been “explained” by aggregation of this peptide within the brain, leaving less to diffuse into the CSF. CSF levels of Aβ42 were later found to correlate inversely with the extent of fibrillar brain amyloid deposition as measured by amyloid PET imaging (64, 65). Although increased levels of CSF tau were present in AD relative to controls, why this occurred was not clear – CSF tau is not a marker of tangle formation, but is increased in situations of significant neuronal damage, for example, after acute stroke (66) or in Creutzfeld–Jacob disease (36). Assays for specifically P-tau also showed increases in AD, and CSF P-tau had higher specificity for AD than did increases of total tau. Only a few studies have tried to identify the forms of tau that are released into CSF. These were found to be N-terminal fragments of tau, with little if any of the full-length protein present (67, 68). The mechanisms of the release of tau into CSF remain unclear. Although converging data across many laboratories and studies have confirmed the profile of decreased Aβ42 and increased total and P-tau in CSF, cutoffs vary across laboratories (63, 69). Extensive quality control efforts have helped to decrease the variability. There are new efforts under way to develop fully automated assays for these key analytes, which will dramatically improve standardization.

Selecting a candidate biomarker has several advantages. Defined biochemical pathways and pathological mechanisms can help to relate the candidate to AD or to another neurodegenerative disorder, which may help to “make sense” of findings regarding the biomarker. Tools for detecting candidate biomarkers may be available, and sensitive detection methods can be developed. As a recent example, tau is released into CSF after neuronal injury. Increased levels of tau can be detected in plasma using ultrasensitive assay methods and were found to be transiently increased in boxers after bouts (70). Post-translational modifications of candidate biomarkers may also be sought and may provide markers related to mechanisms of disease. For example, phosphorylation is important in regulatory and signaling pathways and has been implicated in altering the solubility and promoting aggregation of proteins. P-tau (4) and alpha-synuclein (71) are detectable in CSF and may provide insights into processes relevant to AD and PD, respectively.

Although CSF Aβ42 and tau reflect certain steps of pathology in the brain, much attention has focused on small oligomeric aggregates of these proteins. Evidence suggests that oligomeric forms of Aβ may be the culprits responsible for toxicity (72–74) and also suggests that oligomers and aggregates of tau are species that contribute to neurodegeneration and correlate with cognitive loss in postmortem studies (75, 76). Also, aggregated or oligomeric forms of Aβ and tau may contribute to propagation of pathology (77). Despite the development of sensitive assays that can detect extremely low levels of Aβ oligomers, these have not been consistently or reliably identified in CSF in relation to AD (78, 79).

Several further examples of recent candidate biomarker discovery highlight the continued value of candidate approaches. A candidate approach led to the identification of the neuronal calcium sensor protein visinin-like protein-1 in CSF, and levels were found to be increased in AD relative to controls and predicted progression from non-demented to mild dementia (35) Similarly, a candidate approach was recently used to identify the dendritic protein neurogranin, which is involved in long-term potentiation and calcium regulation, and is decreased and mislocalized in brain tissue in AD. After initial characterization in CSF by HPLC and MS methods an ELISA was developed. Levels of neurogranin were reported to be increased in CSF in AD, even at the stage of MCI (80), and predicted progression from prodromal AD to dementia, as well as rate of progression of MRI change in AD (14). As a second example, genetic studies have implicated variation in the gene that encodes TREM2 as a risk factor in some patients with late-onset AD and later for other neurodegenerative disorders [reviewed in Ref. (81)]. Studies into the biology of cells derived from people homozygous for TREM2 mutations revealed impaired secretion of a cleaved fragment of TREM2. Decreased levels of this fragment were detected using an ELISA in CSF samples from patients with AD (51). Another example is the measurement of levels of granulin to identify people with mutations in the progranulin gene that predisposes to FTD. Progranulin mutations result in haplo-insufficiency and therefore people who carry mutations have a marked decrease in levels of secreted granulin in plasma and CSF (82).

One further example of an important application of CSF biomarkers relates to blood–brain barrier (BBB) integrity. An increased CSF:serum ratio of albumin is an established index used for many years as an indicator of loss of BBB integrity, and together with the IgG index and measurement of myelin basic protein levels, has been used as a diagnostic aid in multiple sclerosis. More recently, other markers of BBB integrity have emerged, particularly in relation to vascular cognitive impairment, and analysis of matrix metalloproteases and neurofilament-light levels have been proposed to supplement the albumin ratio and increase the diagnostic utility for subcortical small vessel disease (40).

A broader targeted approach to discovery is to multiplex known assays in combination [e.g., Luminex panels of assays of secreted proteins; multiple reagent monitoring (MRM) methods to examine selected panels of analytes with spiked in calibrator peptides for quantitation]. Several studies in AD have used arrays or multiplex ELISA-type assays for known secreted proteins to identify biomarkers in plasma and CSF (83, 84). Findings have been inconsistent, and different panels of plasma biomarkers have emerged from different studies, depending on analytical as well as biostatistical methods. Some of the analytes measured in these panels of secreted proteins in CSF showed correlations with cognitive test scores (85), or neuroimaging changes (86) although a validated panel of markers capable of tracking progression in AD has not yet emerged. Data from these studies were used to examine genetic variation associated with CSF levels of 59 proteins, and there were associations for proteins involved in inflammatory signaling (54). There are no validated CSF biomarkers for most non-AD dementias, although patterns of biomarkers, such as CSF P-tau181/total tau ratio, may be helpful in discriminating tauopathies from TDP43-associated FTLD disorders (37).

Targeted biomarker approaches have some disadvantages. Their detection and analysis need specific reagents, e.g., antibodies with high affinity, and antibodies against different regions are typically required to enable quantitative assays to be established and post-translational modifications to be analyzed. Finally, carrying out serial studies of candidate biomarkers and running individual assays to obtain multi-analyte data can be time consuming.

#### Highly Sensitive Assays

Many analytes detectable in plasma or CSF occur at low levels. This can pose a challenge to routine methods of analysis, such as ELISA. Recent technological refinements have resulted in ultrasensitive assay methods, capable of quantitation over low picomolar or femtomolar levels of analytes (87). For example, immuno-PCR, in which an oligonucleotide is conjugated to a detector antibody in a sandwich format, then amplified, has been developed and refined to allowed multiplex assays (88). Another refinement, single molecule arrays (SIMOA), which divides samples into microwells and allows higher detection of signal to background, has been used to identify changes in plasma Aβ in patients who had experienced cardiac arrest (89) and increases in serum or plasma levels of tau in professional athletes after concussion (90), in combat-related traumatic brain injury (TBI) (91), and in patients with major brain trauma (92). Plasma levels of tau are slightly increased in AD compared to controls but are not diagnostically useful (31).The general theme that measuring multiple analytes may paint a more detailed and clearer picture applies to the setting of TBI: recent studies have shown that biomarkers of neuronal, axonal, and astroglial injury appear acutely after the injury, and that axonal markers such as neurofilament protein persist longer in plasma and CSF than markers such as tau (93).

### Non-Targeted Approaches to Protein and Peptide Biomarker Discovery

Non-targeted approaches to biomarker discovery typically involve multiplex and −omic methods, which range from analyzing 10 to 100 analytes to performing large-scale unbiased proteomic or metabolomic screens. These approaches have the advantages of providing coverage of a wide range of potential biomarkers, and of identifying novel markers and mechanisms that may not have been obvious from pathogenic mechanisms or pathology. Also, analyses of interactions between markers, and of how markers relate to biological pathways, can be undertaken. There are several challenges to conducting, analyzing and interpreting large-scale −omic studies. For single analyte assays, a great deal of effort typically goes into development, standardization, and quantitation. By contrast, the analytes in large-scale −omic or similar methods may not be accurately quantified across their dynamic range. Both plasma and CSF have a few dominant proteins, in particular albumin, which are orders of magnitude higher in concentration than the vast majority of proteins and peptides. Methods to deplete the most dominant proteins are often used in −omic studies, but these preparation steps may alter the proteome. It is encouraging that test–retest proteomic analyses after immunodepletion of major proteins in CSF from subjects who underwent repeated lumbar punctures about 1 week apart provided evidence for a reasonably stable proteome (94). Detecting truncated forms of proteins or post-translational modifications may be more difficult in −omic studies using biofluids. Study design and data analysis need to be carefully considered to take proteomic studies from the stage of description or annotation to searching for group differences and the complex series of downstream steps that may lead to identification of candidate peptides and potential markers (95, 96). It is easy to identify false positive biomarker hits when hundreds of potential markers are analyzed and multiple comparisons are made, therefore separate cohorts for discovery and validation are essential. When interpreting findings, it is important to consider what factors may have contributed to the significant group of analytes. For example, vascular disease often coexists with AD, and vascular risk factors may be over-represented in AD patients compared to controls. Especially for proteomic studies of plasma, it is important to take factors such as hypertension, diabetes, weight loss, and decreased physical activity into account during data analyses. As an example of the promise of proteomic studies, recent promising results were reported in a large-scale effort to identify potential biomarkers related to aging through proteomic analysis of plasma, and strategies used in this project are summarized in Ref. (96).

Many non-targeted large-scale proteomic studies of CSF have been conducted in AD. It is interesting to note that Aβ42 and tau have not been detected as AD biomarkers in proteomic analyses of CSF. Early methods of separation, such as 2-dimensional gel electrophoresis (2DGE), resulted in detection and annotation of members of the CSF proteome, but few consistent markers specific for AD appeared. An extension of 2DGE called DIGE uses different fluorescent labels for biosamples from different groups of subjects (e.g., controls and those with disease) and allows for subtle differences to be identified. This has resulted in the discovery of a few novel biomarkers for AD, notably YKL40, a molecule secreted by astrocytes whose levels are increased in CSF in AD (34). MS methods remain the workhorse of proteomics and have been refined and improved in recent years. Analyses of CSF have continued to expand the catalog of proteins detectable in CSF, and a recent study identified and annotated over 2,500 proteins, each identified by at least 2 unique peptides [Ref. (97); database available at http://129.177.231.63/csf-pr/].

Technical improvements in MS have greatly improved the reproducibility of sample runs. Isobaric labeling of peptides, followed by a MS pipeline, can be used to compare samples from different groups of subjects. An approach that resembles the methods used in DIGE yielded several candidate peptide biomarkers for AD (98). Other approaches have allowed targeted quantitative analysis of selected peptides, as well as multiplexing (99, 100). By spiking in samples with heavily labeled known peptides as calibrators, a series of analytes may be analyzed quantitatively, termed MRM or selective reaction monitoring (SRM). For example, an exploratory proteomic study using CSF from patients with familial AD and controls yielded a set of novel candidate biomarkers (101), but these have not been replicated. Another study examined a panel of 39 candidate CSF biomarkers using MRM, and identified 4 that changed over 12 months with progression of AD (102). Recent studies of PD have explored whether a panel of analytes monitored using MRM may have value in diagnosis or relate to cognitive impairment (103). A pipeline for incorporating SRM methods into novel proteomic biomarker discovery has been proposed and its feasibility was demonstrated in a mouse cancer model (104). The sensitivity of MRM is much higher than that of untargeted proteomics, but it still is easier to quantify more abundant proteins, and antibody methods for highly sensitive assays have advantages for lower abundance analytes. Another analytical approach, immunoprecipitation followed by MS, allows differently processed forms of the same protein to be measured in biofluid samples. This targeted approach of MS has been used for the analysis of different forms of Aβ peptides with a variety of different N- and C-terminal amino acids and has provided signatures of the effects of BACE inhibitors on APP processing (60).

Novel approaches to multiplex detection, such as the use of aptamer-based assays or antibody arrays, have allowed the profiling of hundreds to over one thousand analytes simultaneously from small starting volumes of biofluid sample, although the data generated are not truly quantitative (105). Aptamer approaches to screen for plasma biomarkers for AD are under way and have shown some initial promise. For example, in one study, a panel of 13 proteins predicted AD with an area under the ROC curve of 0.7 (30). Other studies that used this technology have found differences between patients with MCI and AD compared to controls, but the specific analytes that were most highly predictive have differed across studies (106, 107). Aptamer technology has also been applied to identify members of the plasma proteome that are changed with aging. In an aging twin study that was followed by replication in several other cohorts, 13 plasma proteins were identified that showed robust changes with aging, some of which are growth factors (108). About 26% of the variability of the markers measured in twins could be explained by a heritable component. Understanding more about the biology of analytes that are detected by aptamer-based tests, and conducting replication studies will be helpful to advance this novel approach to protein biomarker identification.

### Non-Protein and “Unconventional” Biomarkers

Antibodies directed against novel antigens have been sought in serum or plasma as diagnostic markers for AD. Results have not always been consistent, and biomarkers have not yet been established using this method. One approach is to look for antibodies against pathogenic proteins, such as different forms of Aβ, e.g., by screening plasma or serum using micro-arrays. In recent examples, studies that screened for novel conformational forms of pathogenic proteins or unknown antigens that may be diagnostically altered in AD, PD, or other disorders have used auto-antibody and peptoid approaches [e.g., Ref. (32, 33, 109)]. Although initial hits emerged from these studies have not been replicated and the approaches have not yet matured into readily usable assays.

Metabolomic approaches measure small molecules that are substrates or products of metabolic processes. Two analytical methods are typically used, namely MS, which can identify large numbers of metabolites but has slow throughput, and magnetic resonance spectroscopy (MRS), which has higher throughput but lower sensitivity. Several recent small-scale studies have been able to distinguish patterns in CSF samples from AD patients and controls (110, 111). These studies will require extension and replication. Methods to standardize acquisition of metabolomics data are needed in order for these to be able to be readily used by reference laboratories. Increased statistical rigor and the need for extensive replication strongly need to be applied to metabolomic studies (112). Lipidomic analyses have also been applied to AD, with inconsistent findings. One recent study identified a panel of lipid-related biomarkers in plasma that predicted conversion to AD (29). Although clinical assessment, sample handling, and biomarker analysis were carefully standardized in this study, the number of subjects who progressed from normal cognition to impairment was small. This panel of biomarkers has not yet been replicated. Another lipidomic study identified changes in long chain cholesteryl esters in plasma that discriminated patients with AD and controls, but lacked replication cohorts (113). Careful study design with large enough numbers and replication cohorts are essential to make progress in this area. Also, robust assay platforms will need to be developed that will allow a set of lipidomic assays to be routinely run as a mature assay.

Exosomes are a subset of microvesicles and are released from cells under physiological and pathological conditions and circulate in body fluids. Exosomes are smaller than microparticles, and are usually defined as <100 nM in diameter. This small size poses a challenge to current methods of detection using flow cytometry. Exosomes arise from intracellular microvesicular bodies, whereas microparticles originate from the plasma membranes of cells or from apoptotic bodies. Exosomes may be implicated in neurodegenerative disorders in altered intercellular communication, for example, by transporting microRNA (miRNA), or by contributing to the spread of misfolded proteins (114). Methods to isolate exosomes have not been well standardized, and commercial kits yield mixed populations of exosomes and other particles. Extracellular vesicles, including exosomes, are found in CSF and their proteome has been characterized (115, 116). To date, there are no clear diagnostic markers that distinguish AD based on CSF exosomes, but much work is ongoing. Recent reports have isolated and analyzed exosomes in plasma, after using an immunopurification step to isolate a subset that have surface markers suggesting their neuronal origin, such as L1 cellular adhesion molecule (L1CAM) (117, 118). Subsequent protein analyses using ELISA identified differences in levels of AD protein biomarkers of Aβ42 and tau (118) between AD and controls. These are promising initial findings, but much further work is needed to replicate and extend the findings. For example, it is unclear how exosomes might traffic from the CNS to the bloodstream, and therefore whether these truly reflect neuronal pathophysiology. Also, the multiple steps necessary to isolate exosomes and then assay their contents poses challenges to assay standardization.

MicroRNAs are small RNA species that control gene expression by binding to sets of target mRNAs and may play roles in intracellular communication. They can be isolated from exosomes or directly from biofluids. There are technical problems in quantifying levels of miRNAs, and the development of methods and standards are still in their early stages. Studies in AD have identified profiles of miRNAs in CSF that may distinguish patients from controls but have been inconsistent across studies (119–121). Levels of miRNA levels are affected by the presence of cells, so that careful standardization will be necessary for studies using CSF (121). Studies of miRNA are reviewed in more detail in this collection of reviews (122).

Peripheral cells, such as mononuclear cells and lymphocytes, as well as platelets have been the subjects of many types of biomarker studies in AD. The nature of these studies and the types of biomarkers that have been sought are too diverse to be easily summarized here. Although an enormous number of markers and biological processes can be interrogated using cells, to date, no consistent biomarker profiles have emerged that were subsequently widely replicated.

## Validating and Understanding Biomarkers

The initial validation of biomarkers requires the development of quantitative, sensitive, and reliable assays, and identifying pre-analytical and analytical factors that may influence the levels that are measured (123). As examples of pre-analytical factors, for Aβ, polypropylene collection tubes are required, whereas for alpha-synuclein, measuring the extent of contamination by hemoglobin is important (124). Effects of storage, freeze–thaw cycles, and sample handling need to be carefully determined. Assay performance metrics, the type of analytical platform to be used, preparation and use of analytical standards and biological replicates also need to be standardized. Appropriately scaled clinical studies aimed at determining cutoff points, sensitivity, and specificity need to be conducted. Depending on the proposed use of the biomarker, longitudinal studies and postmortem confirmation of pathological features of brain pathology may add credence to claims for sensitivity and specificity. Meta-analyses or pooled analyses of multi-center data can provide information about effects of age and APOE genotype on CSF biomarkers (65). Assays typically progress through different stages of qualification. Much effort has gone into comparisons of A-beta and tau assays, including round robin efforts, which also were recently applied to MS assays for A-beta (125), and international quality control efforts. Next-generation assays for A-beta42, tau, and P-tau may help to decrease variability and to develop rigorous and standardized cutoff points that are readily applicable across laboratories. Understanding the phenomena that the biomarkers are measuring goes beyond these validation steps that have been outlined, and it is a critical step in determining the use of biomarkers, particularly regarding therapeutic studies. As a sobering observation, although increased CSF levels of tau and P-tau are routinely detected in AD, the mechanisms whereby these biomarkers are released into the CSF are not well understood.

There are many opportunities to study genetics in relation to biomarkers, some of which have been discussed earlier. Large-scale studies of patients with inherited forms of early onset AD are helping to expand the map and timeline of biomarkers (126). Because age is the strongest risk factor for sporadic AD, it is important to continue to study how biomarkers and related brain processes change during aging. As an example, studies of Aβ metabolism using SILK have shown that there are marked changes in parameters related to production and clearance of Aβ from the CSF in association with aging (127).

## Toward an Expanded Suite of Biomarkers

Biomarkers in biofluids have provided several important insights into AD, and currently have a role both in diagnosis and in the development of therapy. An attainable future goal is to improve and standardize current assays for Aβ, tau, and P-tau to permit routine and widespread clinical use. Progress will continue to be made in the development of assays to allow early and pre-symptomatic detection of AD to facilitate therapeutic studies (128). An ambitious goal will be to identify biomarkers that predict who is at risk for beginning to developing amyloid deposition in the brain before these deposits arise. In the area of diagnostics, the development of multi-analyte panels that are able to provide indices of non-AD degenerative disorders and important biological processes will remain an important area of research. As an illustrative example, a recent study of a nine analyte panel of CSF biomarkers had good differential diagnostic ability to distinguish between atypical movement disorders, PD and AD (129).

For clinical trials, a suite of biomarkers to evaluate amyloid processing exists, but markers related to oligomers remain elusive. Biomarkers that inform about target engagement for other therapeutic areas, for example, tau therapeutics, require further development. Prognostic, predictive, and companion biomarkers have not yet been identified and can be sought in the context of longitudinal studies. Relationships between biofluid markers, brain imaging, and cognitive testing will help to refine the roadmap of progression along the way to dementia in AD, especially during preclinical and prodromal stages. The potential for plasma biomarkers to provide screening, diagnostic, or prognostic tools merits continued study, but the design and validation of a plasma biomarker may be more complex than for a CSF biomarker.

The growth of research and development of new technologies gives hope that we may be able to develop a more comprehensive suite of biomarkers to build a detailed picture of the brain, that may integrate markers related to different cell types, important cellular structures such as synapses, biological processes such as transport, lipid metabolism, and exosome release, and effects of damage, oxidative stress, and inflammation. Progress in these areas holds the promise of greatly extending the reach of biofluid biomarkers for AD and related disorders.

## Conflict of Interest Statement

The author declares that the research was conducted in the absence of any commercial or financial relationships that could be construed as a potential conflict of interest.
